# Poly[diaqua-μ_2_-oxalato-di-μ_2_-pyrimidine-2-carboxyl­ato-dimanganese(II)]

**DOI:** 10.1107/S1600536808002687

**Published:** 2008-04-02

**Authors:** Antonio Rodríguez-Diéguez, Hakima Aouryaghal, A. J. Mota, Enrique Colacio

**Affiliations:** a Departamento de Química Inorgánica, Facultad de Ciencias, Universidad de Granada, c/ Severo Ochoa s/n, 18071 Granada, Spain; b Departement de Chimie, Université Abdelmalek Essaadi, Faculté de Sciences, PO 2121, Tétouan, Morocco

## Abstract

In the title compound, [Mn_2_(C_2_O_4_)(C_5_H_3_N_2_O_2_)_2_(H_2_O)_2_]_*n*_, the Mn^II^ atom exhibits a distorted octa­hedral coordination geometry, with the centrosymmetric oxalate anion and the monoanionic pyrimidine-2-carboxyl­ate ligands generating a two-dimensional honeycomb network with a (6,3)-topology.

## Related literature

For the preparation of 2-cyano­pyrimidine, see: Rodríguez-Diéguez, Salinas-Castillo *et al.* (2007 ▶). For related literature, see: Rodríguez-Diéguez, Cano *et al.* (2007 ▶). 
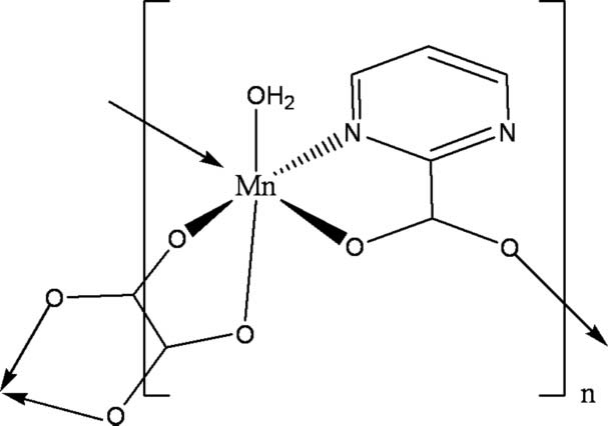


## Experimental

### 

#### Crystal data

[Mn_2_(C_2_O_4_)(C_5_H_3_N_2_O_2_)_2_(H_2_O)_2_]
*M* *_r_* = 480.12
Monoclinic, 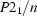

*a* = 7.5447 (7) Å
*b* = 11.1944 (11) Å
*c* = 9.7259 (10) Å
β = 102.4220 (10)°
*V* = 802.20 (14) Å^3^
*Z* = 2
Mo *K*α radiation
μ = 1.64 mm^−1^
*T* = 150 (2) K
0.22 × 0.21 × 0.20 mm

#### Data collection

Bruker SMART APEX CCD area-detector diffractometer
Absorption correction: multi-scan (*SADABS*; Sheldrick, 2004 ▶) *T*_min_ = 0.714, *T*_max_ = 0.773 (expected range = 0.665–0.720)
5847 measured reflections
1495 independent reflections
1389 reflections with *I* > 2σ(*I*)
*R*_int_ = 0.019

#### Refinement

*R*[*F* ^2^ > 2σ(*F* ^2^)] = 0.025
*wR*(*F* ^2^) = 0.062
*S* = 1.11
1495 reflections
127 parameters
H-atom parameters constrained
Δρ_max_ = 0.41 e Å^−3^
Δρ_min_ = −0.21 e Å^−3^

### 

Data collection: *SMART* (Bruker, 2001 ▶); cell refinement: *SAINT* (Bruker, 2001 ▶); data reduction: *SAINT*; program(s) used to solve structure: *SHELXTL* (Sheldrick, 2008 ▶); program(s) used to refine structure: *SHELXTL*; molecular graphics: *SHELXTL*; software used to prepare material for publication: *publCIF* (Westrip, 2008 ▶).

## Supplementary Material

Crystal structure: contains datablocks global, I. DOI: 10.1107/S1600536808002687/su2042sup1.cif

Structure factors: contains datablocks I. DOI: 10.1107/S1600536808002687/su2042Isup2.hkl

Additional supplementary materials:  crystallographic information; 3D view; checkCIF report

## Figures and Tables

**Table 1:** Hydrogen-bond geometry (Å, °)

*D*—H⋯*A*	*D*—H	H⋯*A*	*D*⋯*A*	*D*—H⋯*A*
O1*W*—H2*WB*⋯O2*B*^i^	0.80	2.05	2.847 (2)	170
O1*W*—H1*WA*⋯N5^ii^	0.77	2.05	2.815 (2)	171

Symmetry codes: (i) 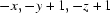; (ii) 
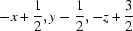
.

**Fig. 1.**
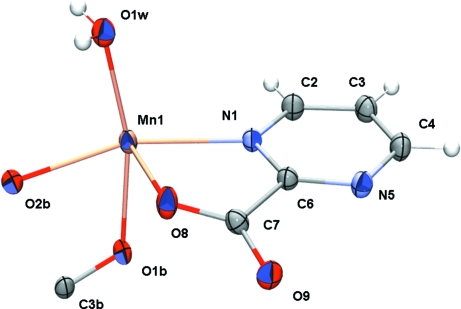
The molecular structure of the asymmetric unit of [Mn2(pymca)2(ox)(H2O)2]n, showing the atom labels. Thermal ellipsoids are drawn at the 50% probability level. H atoms are represented as spheres of arbitrary radii.

**Fig. 2.**
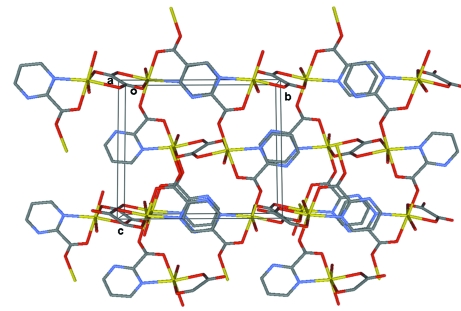
A view down the a axis of the crystal structure of [Mn2(pymca)2(ox)(H2O)2]n, showing the environment of the manganese atoms and the bidimensional (6,3) net topology. The H atoms have been omitted for clarity.

## supplementary crystallographic information

### Comment

The title compound constitutes a new member of a series of honeycomb type compounds previously reported by us (Rodríguez-Diéguez, Cano *et al.*, 2007).

The asymmetric unit of the title compound is illustrated in Fig. 1. The Mn(II) atom exhibits a distorted octahedral coordination geometry built by one pyrimidine-2-carboxylato ligand, half of an oxalic acid ligand and one water molecule. The compound can be described by Mn(pyrimidine-2-carboxylato) chains linked by oxalate ligands to obtain a bidimensional coordination polymer. Each Mn(II) is connected to three Mn atoms through two pyrimidine-2-carboxylato ligands and one oxalate ligand, generating a two-dimensional honeycomb network with a (6,3) topology (Fig. 2). The shortest perpendicular distance between symmetry related pyrimidine rings is *ca* 3.41 Å.

### Experimental

The multitopic bridging ligand 2-carboxy-pyrimidine (H-pymca) was prepared by basic hydrolysis of 2-cyanopyrimidine with KOH and further neutralization with 2 N HCl. The title compound was obtained by the reaction of a mixture of two solutions. The first contained pyrimidine-2-carboxylato (17.1 mg) and MnCl_2_.4(H_2_O) (8.67 mg) in water/MeOH (10 ml). The second was formed by addition of MnCl_2_.4(H_2_O) (8.67 mg) to a solution of sodium oxalate (9.23 mg) in water (10 ml). These two solutions were then mixed and stirred for 2 h to give a pale-yellow solution. After standing at room temperature for several days prismatic yellow crystals appeared.

### Refinement

The water H atoms were located in a difference Fourier map and refined as riding atoms with O—H = 0.77 and 0.80 Å and *U*_iso_(H) = 1.2*U*_eq_(O). The pyrimidine H atoms were positioned geometrically and treated as riding atoms with C—H = 0.93 Å, and *U*_iso_(H) = 1.2*U*_eq_(C).

### Crystal data

**Table:**

[Mn_2_(C_2_O_4_)(C_5_H_3_N_2_O_2_)_2_(H_2_O)_2_]	*F*_000_ = 480
*M**_r_* = 480.12	*D*_x_ = 1.988 Mg m^−^^3^
Monoclinic, *P*2_1_/*n*	Mo *K*α radiation λ = 0.71073 Å
Hall symbol: -P 2yn	Cell parameters from 3384 reflections
*a* = 7.5447 (7) Å	θ = 2.8–28.9º
*b* = 11.1944 (11) Å	µ = 1.64 mm^−^^1^
*c* = 9.7259 (10) Å	*T* = 150 (2) K
β = 102.4220 (10)º	Prismatic, yellow
*V* = 802.20 (14) Å^3^	0.22 × 0.21 × 0.20 mm
*Z* = 2	

### Data collection

**Table:**

Bruker SMART APEX CCD area-detector diffractometer	1389 reflections with *I* > 2σ(*I*)
Monochromator: graphite	*R*_int_ = 0.019
*T* = 150(2) K	θ_max_ = 25.5º
φ and ω scans	θ_min_ = 2.8º
Absorption correction: multi-scan(SADABS; Sheldrick, 2004)	*h* = −9→9
*T*_min_ = 0.714, *T*_max_ = 0.774	*k* = −13→13
5847 measured reflections	*l* = −11→11
1495 independent reflections	

### Refinement

**Table:**

Refinement on *F*^2^	Secondary atom site location: difference Fourier map
Least-squares matrix: full	Hydrogen site location: inferred from neighbouring sites
*R*[*F*^2^ > 2σ(*F*^2^)] = 0.025	H-atom parameters constrained
*wR*(*F*^2^) = 0.062	*w* = 1/[σ^2^(*F*_o_^2^) + (0.0274*P*)^2^ + 0.6516*P*] where *P* = (*F*_o_^2^ + 2*F*_c_^2^)/3
*S* = 1.11	(Δ/σ)_max_ < 0.001
1495 reflections	Δρ_max_ = 0.41 e Å^−^^3^
127 parameters	Δρ_min_ = −0.21 e Å^−^^3^
Primary atom site location: structure-invariant direct methods	Extinction correction: none

### Special details

**Table:**

Geometry. All e.s.d.'s (except the e.s.d. in the dihedral angle between two l.s. planes) are estimated using the full covariance matrix. The cell e.s.d.'s are taken into account individually in the estimation of e.s.d.'s in distances, angles and torsion angles; correlations between e.s.d.'s in cell parameters are only used when they are defined by crystal symmetry. An approximate (isotropic) treatment of cell e.s.d.'s is used for estimating e.s.d.'s involving l.s. planes.
Refinement. Refinement of *F*^2^ against ALL reflections. The weighted *R*-factor *wR* and goodness of fit *S* are based on *F*^2^, conventional *R*-factors *R* are based on *F*, with *F* set to zero for negative *F*^2^. The threshold expression of *F*^2^ > σ(*F*^2^) is used only for calculating *R*-factors(gt) *etc*. and is not relevant to the choice of reflections for refinement. *R*-factors based on *F*^2^ are statistically about twice as large as those based on *F*, and *R*- factors based on ALL data will be even larger.

### Fractional atomic coordinates and isotropic or equivalent isotropic displacement parameters (Å^2^)

**Table:**

	*x*	*y*	*z*	*U*_iso_*/*U*_eq_	
Mn1	0.22294 (4)	0.67224 (3)	0.51301 (3)	0.01810 (12)	
N1	0.2376 (2)	0.87496 (16)	0.49706 (17)	0.0170 (4)	
C2	0.1555 (3)	0.94939 (19)	0.3937 (2)	0.0224 (5)	
H2	0.0850	0.9174	0.3117	0.027*	
C3	0.1735 (3)	1.07187 (19)	0.4065 (2)	0.0230 (5)	
H3	0.1164	1.1230	0.3353	0.028*	
C4	0.2805 (3)	1.1150 (2)	0.5301 (2)	0.0229 (5)	
H4	0.2929	1.1972	0.5428	0.027*	
N5	0.3670 (2)	1.04205 (16)	0.63259 (18)	0.0196 (4)	
C6	0.3405 (3)	0.92559 (18)	0.6111 (2)	0.0166 (4)	
C7	0.4354 (3)	0.83993 (18)	0.7255 (2)	0.0182 (4)	
O8	0.3827 (2)	0.73395 (13)	0.71406 (16)	0.0251 (4)	
O9	0.5576 (2)	0.88282 (13)	0.81900 (15)	0.0224 (3)	
O1B	0.4755 (2)	0.64691 (13)	0.43885 (16)	0.0230 (3)	
O2B	0.30121 (19)	0.48353 (13)	0.56335 (16)	0.0217 (3)	
C3B	0.5504 (3)	0.54766 (18)	0.4641 (2)	0.0185 (4)	
O1W	−0.0119 (2)	0.64879 (13)	0.60530 (15)	0.0219 (3)	
H2WB	−0.0848	0.6091	0.5523	0.026*	
H1WA	0.0167	0.6192	0.6778	0.026*	

### Atomic displacement parameters (Å^2^)

**Table:**

	*U*^11^	*U*^22^	*U*^33^	*U*^12^	*U*^13^	*U*^23^
Mn1	0.01820 (19)	0.01263 (18)	0.01991 (19)	0.00050 (12)	−0.00384 (13)	−0.00014 (12)
N1	0.0170 (9)	0.0150 (9)	0.0171 (9)	0.0001 (7)	−0.0008 (7)	−0.0001 (6)
C2	0.0235 (11)	0.0219 (11)	0.0194 (10)	−0.0003 (9)	−0.0009 (9)	0.0000 (9)
C3	0.0250 (11)	0.0198 (11)	0.0229 (11)	0.0005 (9)	0.0024 (9)	0.0033 (9)
C4	0.0259 (11)	0.0165 (11)	0.0257 (11)	−0.0010 (9)	0.0044 (9)	0.0013 (9)
N5	0.0219 (9)	0.0166 (9)	0.0187 (9)	−0.0014 (7)	0.0007 (7)	−0.0005 (7)
C6	0.0162 (10)	0.0162 (10)	0.0170 (10)	0.0002 (8)	0.0026 (8)	−0.0002 (8)
C7	0.0185 (10)	0.0184 (11)	0.0167 (10)	0.0026 (8)	0.0016 (8)	−0.0003 (8)
O8	0.0297 (9)	0.0157 (8)	0.0241 (8)	−0.0014 (6)	−0.0074 (7)	0.0027 (6)
O9	0.0222 (8)	0.0211 (8)	0.0195 (8)	−0.0023 (6)	−0.0054 (6)	0.0001 (6)
O1B	0.0223 (8)	0.0136 (7)	0.0316 (8)	0.0020 (6)	0.0028 (7)	0.0050 (6)
O2B	0.0188 (7)	0.0163 (7)	0.0282 (8)	0.0010 (6)	0.0009 (6)	0.0016 (6)
C3B	0.0172 (10)	0.0151 (10)	0.0191 (10)	−0.0009 (8)	−0.0055 (8)	−0.0017 (8)
O1W	0.0235 (8)	0.0196 (8)	0.0193 (7)	−0.0002 (6)	−0.0028 (6)	0.0028 (6)

### Geometric parameters (Å, °)

**Table:**

Mn1—O9^i^	2.1175 (14)	C4—H4	0.9300
Mn1—O1W	2.1677 (15)	N5—C6	1.329 (3)
Mn1—O8	2.1771 (15)	C6—C7	1.526 (3)
Mn1—O1B	2.1958 (16)	C7—O9	1.244 (3)
Mn1—O2B	2.2196 (15)	C7—O8	1.248 (3)
Mn1—N1	2.2790 (18)	O9—Mn1^ii^	2.1175 (14)
N1—C6	1.336 (3)	O1B—C3B	1.247 (2)
N1—C2	1.349 (3)	O2B—C3B^iii^	1.255 (3)
C2—C3	1.381 (3)	C3B—O2B^iii^	1.255 (3)
C2—H2	0.9300	C3B—C3B^iii^	1.560 (4)
C3—C4	1.383 (3)	O1W—H2WB	0.8027
C3—H3	0.9300	O1W—H1WA	0.7669
C4—N5	1.344 (3)		
			
O9^i^—Mn1—O1W	87.50 (6)	C2—C3—H3	121.5
O9^i^—Mn1—O8	177.38 (6)	C4—C3—H3	121.5
O1W—Mn1—O8	90.61 (6)	N5—C4—C3	122.1 (2)
O9^i^—Mn1—O1B	93.21 (6)	N5—C4—H4	118.9
O1W—Mn1—O1B	164.73 (6)	C3—C4—H4	118.9
O8—Mn1—O1B	89.08 (6)	C6—N5—C4	116.55 (18)
O9^i^—Mn1—O2B	89.85 (6)	N5—C6—N1	126.02 (19)
O1W—Mn1—O2B	89.76 (6)	N5—C6—C7	118.09 (18)
O8—Mn1—O2B	91.96 (5)	N1—C6—C7	115.90 (18)
O1B—Mn1—O2B	74.99 (5)	O9—C7—O8	127.13 (19)
O9^i^—Mn1—N1	104.89 (6)	O9—C7—C6	116.64 (18)
O1W—Mn1—N1	101.81 (6)	O8—C7—C6	116.22 (18)
O8—Mn1—N1	73.72 (6)	C7—O8—Mn1	119.06 (13)
O1B—Mn1—N1	92.76 (6)	C7—O9—Mn1^ii^	137.83 (14)
O2B—Mn1—N1	161.49 (6)	C3B—O1B—Mn1	116.05 (14)
C6—N1—C2	116.64 (18)	C3B^iii^—O2B—Mn1	115.15 (13)
C6—N1—Mn1	113.21 (13)	O1B—C3B—O2B^iii^	126.4 (2)
C2—N1—Mn1	130.12 (14)	O1B—C3B—C3B^iii^	117.0 (2)
N1—C2—C3	121.7 (2)	O2B^iii^—C3B—C3B^iii^	116.6 (2)
N1—C2—H2	119.2	Mn1—O1W—H2WB	108.0
C3—C2—H2	119.2	Mn1—O1W—H1WA	109.9
C2—C3—C4	117.0 (2)	H2WB—O1W—H1WA	111.7

Symmetry codes: (i) *x*−1/2, −*y*+3/2, *z*−1/2; (ii) *x*+1/2, −*y*+3/2, *z*+1/2; (iii) −*x*+1, −*y*+1, −*z*+1.

### Hydrogen-bond geometry (Å, °)

**Table:**

*D*—H···*A*	*D*—H	H···*A*	*D*···*A*	*D*—H···*A*
O1W—H2WB···O2B^iv^	0.80	2.05	2.847 (2)	170
O1W—H1WA···N5^v^	0.77	2.05	2.815 (2)	171

Symmetry codes: (iv) −*x*, −*y*+1, −*z*+1; (v) −*x*+1/2, *y*−1/2, −*z*+3/2.
